# The Interplay of Comorbidities in Chronic Heart Failure: Challenges and Solutions

**DOI:** 10.2174/011573403X289572240206112303

**Published:** 2024-02-09

**Authors:** Shashipriya Agress, Jannat S. Sheikh, Aida A. Perez Ramos, Durlav Kashyap, Soha Razmjouei, Joy Kumar, Mankaranvir Singh, Muhammad Ali Lak, Ali Osman, Muhammad Zia ul Haq

**Affiliations:** 1 Government Medical College, Kadapa, India;; 2 CMH Lahore Medical College & Institute of Dentistry, Lahore, Pakistan;; 3 Universidad Central de Venezuela, Caracas, Venezuela;; 4 West China Medical School, Sichuan University, Chengdu, China;; 5 Case Western Reserve University, Cleveland, OH, United States of America;; 6 Kasturba Medical College, Manipal, India;; 7 Government Medical College, Patiala, Punjab, India;; 8 Department of Internal Medicine, CMH Lahore Medical College & Institute of Dentistry, Lahore, Pakistan;; 9 Faculty of Medicine, University of Khartoum, Khartoum, Sudan;; 10 Department of Epidemiology and Public Health, Emory University Rollins School of Public Health, Atlanta, USA;; 11 Department of Noncommunicable Diseases and Mental Health, World Health Organization, Cairo, Egypt

**Keywords:** Chronic heart failure, comorbidity management, multidisciplinary approach, reduced ejection fraction, preserved ejection fraction, treatment standardization

## Abstract

**Background:**

Chronic heart failure (HF) is frequently associated with various comorbidities. These comorbid conditions, such as anemia, diabetes mellitus, renal insufficiency, and sleep apnea, can significantly impact the prognosis of patients with HF.

**Objective:**

This review aims to synthesize current evidence on the prevalence, impact, and management of comorbidities in patients with chronic HF.

**Methods:**

A comprehensive review was conducted, with a rigorous selection process. Out of an initial pool of 59,030 articles identified across various research modalities, 134 articles were chosen for inclusion. The selection spanned various research methods, from randomized controlled trials to observational studies.

**Results:**

Comorbidities are highly prevalent in patients with HF and contribute to increased hospitalization rates and mortality. Despite advances in therapies for HF with reduced ejection fraction, options for treating HF with preserved ejection fraction remain sparse. Existing treatment protocols often lack standardization, reflecting a limited understanding of the intricate relationships between HF and associated comorbidities.

**Conclusion:**

There is a pressing need for a multidisciplinary, tailored approach to manage HF and its intricate comorbidities. This review underscores the importance of ongoing research efforts to devise targeted treatment strategies for HF patients with various comorbid conditions.

## 1 INTRODUCTION

Heart failure (HF) is a prevalent cardiovascular disorder characterized by the heart's inability to circulate blood effectively, resulting in reduced cardiac output. This dysfunction can precipitate a cascade of complications, including fluid retention, weakening of the cardiac muscle, and arrhythmias, leading to compromised cardiac function [1]. A recent study revealed that the global incidence of HF nearly doubled from 33.5 million in 1990 to 64.3 million in 2017, accompanied by a 29.5% increase in age-standardized prevalence rates, particularly in regions, such as Oceania, Central Asia, and Eastern Europe. Cardiovascular diseases (CVDs), including HF, account for 32% of global deaths, according to the World Health Organization [2]. However, in industrialized nations, the prevalence of HF is stabilizing or even declining due to advances in treatment and an aging population [3].

HF has several common etiologies, including coronary artery disease, hypertension, and valvular abnormalities. It manifests symptomatically as dyspnea, orthopnea, edema, and elevated jugular venous pressure, among others [4].

Acute and chronic forms of HF are recognized, with acute HF characterized by a sudden decline in cardiac function and chronic HF (CHF) developing more gradually. Regardless of the form, both are associated with similar symptoms, and acute HF is particularly prevalent among the elderly, with higher mortality rates [5, 6].

Significantly, HF often presents with multiple non-cardiovascular comorbidities, which complicates its clinical management [7, 8]. Common comorbidities include renal insufficiency, diabetes mellitus, sleep apnea, chronic obstructive pulmonary disease (COPD), and anemia, which are associated with increased hospitalization and mortality [9].

Despite advancements in HF therapeutics, the management of these comorbid conditions often remains suboptimal. Current guidelines are insufficient, primarily due to evidence gaps and gender disparities in clinical trials [9, 10].

In this comprehensive review, we aim to synthesize existing literature on HF comorbidities, paying particular attention to the gaps in methodological rigor and the scarcity of randomized controlled trials (RCTs). Our ultimate objective is to clarify the complex relationship between HF and its comorbid conditions, assess the efficacy of existing pharmacotherapies, and refine clinical guidelines. By doing so, we aspire to inform future research that improves both prognostic outcomes and quality of life for HF patients with comorbidities.

## 2 METHODOLOGY

We designed a rigorous methodology to analyze the extant literature on comorbidities in chronic HF critically. Utilizing PubMed and Google Scholar, we performed an exhaustive literature search guided by keywords including “Heart Failure,” “Comorbidities,” and specific comorbid conditions such as “Anemia” and “Diabetes Mellitus”.

Inclusion criteria were stringent: we focused on English-language articles published in the past decade, encompassing a range of study designs-systematic reviews, meta-analyses, RCTs, and both prospective and retrospective observational studies. Eligible studies had to examine human subjects with chronic HF and one or more comorbidities and must have concentrated on pharmacological interventions for these conditions.

We set explicit exclusion criteria to uphold the review's integrity. Non-research-based articles, studies not focusing on human subjects with HF, and works published in languages other than English were excluded. We also dismissed preclinical studies involving only animal models or *in vitro* analyses.

**Fig. (1):**
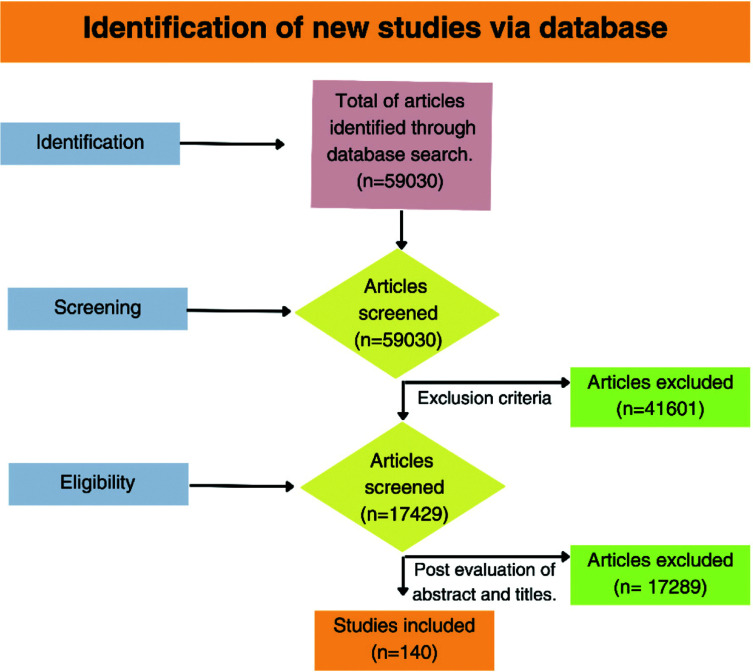
Illustrative flowchart depicting the article selection process for the comprehensive review. This diagram is representative of the rigorous methodology employed to curate relevant literature and is not indicative of a systematic review as defined by PRISMA guidelines.

The checklist provided by Scale for the Assessment of Narrative Review Articles (SANRA) served as the basis for the quality assessment. The PRISMA guidelines provided guidance for the study selection process, but due to the wider scope of our review, it was not restricted by them [11]. Our initial search yielded 54,658 articles; rigorous screening reduced this to 17,193 for detailed evaluation. After further scrutiny, 117 articles met all criteria and were included in the final review (Fig. **1**).

This methodologically robust approach not only informs evidence-based clinical practice but also identifies critical gaps in the existing research landscape, thereby laying the groundwork for future investigations. This approach, while inspired by the systematic nature of review methodologies, was adapted to meet the objectives of a comprehensive review and thus did not follow the systematic review protocol typically associated with a PRISMA checklist.

## 3 PREVALENCE AND IMPACT OF COMORBIDITIES IN HF

### 3.1 Anemia

Anemia as defined by the World Health Organization (WHO) criteria: Hemoglobin levels below 12.0 g/L in women and 13.0 g/L in men [12, 13]. Retrospective studies have reported that up to 41.9% of HF patients exhibit anemia [14]. Further, its prevalence varies across ejection fraction categories- 41% in heart failure with preserved ejection fraction (HFpEF) patients and 32% in heart failure with reduced ejection fraction (HFrEF) patients [13].

In HF, anemia often stems from iron deficiency, related to low ferritin levels and elevated hepcidin, which impairs iron release [15]. Various other factors-including age, coronary artery disease, chronic renal disease, atrial fibrillation, gender, diabetes, and high systolic blood pressure, contribute to the development of anemia in HF [16].

The pathophysiology of anemia in HF involves complex mechanisms such as impaired erythropoietin production, cytokine activation, and reduced renal blood flow [16]. In acute heart failure (AHF), the etiology is multifactorial, influenced by volume overload, renal dysfunction, malnutrition, and certain medications [17].

Notably, anemia worsens HF by lowering systemic vascular resistance, activating the renin-angiotensin-aldosterone system (RAAS), and promoting salt and water retention as depicted in Fig. (**2**) [16]. Patients with HF and anemia face higher morbidity and mortality, underscoring the critical need for early identification and treatment [18]. Intravenous (IV) iron delivery has proven effective for HF patients with iron deficiency, as endorsed by both the 2017 AHA and the 2016 European Society of Cardiology (ESC) guidelines [19].

### 3.2 Diabetes Mellitus

DM and HF often coexist, with approximately one-third of HF patients also having DM; hence, it's an important risk factor [20]. A post-hoc analysis of a randomized, double-blind, multi-center trial comparing sacubitril/valsartan to valsartan alone revealed that 38% of the 4796 HFpEF patients had DM, while 28% had pre-diabetes [21].

Various studies have delineated distinctive phenotypic markers in diabetic patients. Compared to individuals with normal glycated hemoglobin levels (HbA1c), diabetics tend to have higher BMI, waist circumference, and waist-hip ratio. They also present higher rates of atherosclerotic disease, hypertension, anemia, and sleep apnea [21]. Furthermore, diabetics manifest lower atrial fibrillation rates, reduced hemoglobin levels, and compromised renal function. Although echocardiographic differences were not apparent, cardiac magnetic resonance (CMR) disclosed increased left ventricular mass and significant myocardial fibrosis in diabetics [22].

DM exerts a potent influence on HF progression, as evidenced by a prospective cohort analysis using data from the Atherosclerosis Risk in Communities (ARIC) study. This study stratified 4774 adults into preclinical HF stages A or B according to the 2021 Universal Definition and found that uncontrolled DM (HbA1c >7%) was strongly associated with progression to overt HF, particularly in individuals at preclinical stage B [23].

Hospitalization rates and lengths of stay are also notably elevated in HF patients with DM. A UK cohort study examined 711 individuals with stable HF and HFrEF, 32% of whom had DM. Over a 4-year follow-up, DM patients had higher annual hospital admission rates and longer stays, primarily due to decompensated HF, other cardiovascular events, and infections [24].

Both diabetics and prediabetics exhibit worse New York Heart Association (NYHA) class distribution and KQCQ clinical summary scores, yet their plasma NT-proBNP levels are comparable to those with normal HbA1c [21]. DM is associated with increased all-cause mortality in AHF patients, particularly those with HFrEF [25]. Given DM's substantial impact on HF, it's crucial to emphasize early detection and a multidisciplinary management approach [24].

The dearth of data on interaction mechanisms suggests a need for further studies aimed at clarifying the etiology and characteristics of HF in diabetics and fostering the development of effective prevention and treatment strategies.

Diabetes not only affects blood glucose regulation but also poses a considerable risk for HF due to processes like macrovasculopathy, microvasculopathy, and vasculopathy-independent myocardial dysfunction [26]. Hyperglycemia, insulin resistance, and hyperinsulinemia seem to be the driving forces behind these detrimental changes. Both direct myocardial impact and indirect vasculopathic effects contribute to the pathology [26].

Macrovasculopathy involves endothelial and vascular smooth muscle cell (VSMC) dysfunction, disrupting vascular homeostasis and leading to pro-inflammatory/thrombotic states, thereby precipitating cardiovascular disease and potential HF [26]. In microvasculopathy, aberrant endothelial cell signaling narrows capillary diameter, exacerbating vascular dysfunction at the capillary level [26].

Vasculopathy-independent myocardial dysfunction results from altered glucose metabolism and a shift toward less efficient fatty acid oxidation. These metabolic changes have the potential to initiate and worsen cardiac dysfunction, culminating in HF [27].

### 3.3 Renal Disease

Renal dysfunction (RD) commonly coexists with HF and significantly impacts patient prognosis. One study demonstrated that among 606 patients, severe RD (eGFR <30 mL/min/1.73 m^2^) was present in 23.8%, moderate RD (eGFR 30-<60 mL/min/1.73 m^2^) in 53.3%, and no RD (eGFR ≥60 mL/min/1.73 m^2^) in 22.9%. Moreover, end-stage renal disease (ESRD) was evident in 6.1% of these patients [28]. In HF patients with mitral regurgitation, particularly those treated with Mitral clip plus GDMT, preexisting severe RD elevated the two-year mortality risk, which further escalated if ESRD developed [28].

A crucial investigation evaluated the influence of chronic kidney disease (CKD) on the progression of structural damage in HF patients over a time of 8 years, finding that this group presented more frequently with left ventricular adverse changes that caused decreased ejection fraction. CKD-enabled structural alterations is considered an independent risk factor for hospitalization and death [29]. Another research indicated that the urinary-to-serum creatinine ratio (UC/SC) serves as a valuable prognostic marker, with intrinsic kidney dysfunction correlating with the highest rate of cardio-renal events [30]. Indeed, every 10 mL/min/1.73 m^2 decline in eGFR corresponds to a 12% increase in mortality risk [31].

The kidneys receive 20-25% of cardiac output, making them particularly vulnerable to hemodynamic shifts caused by HF. These changes can result in cardiorenal syndrome (CRS), activated by compensatory mechanisms such as the renin-angiotensin-aldosterone system (RAAS), sympathetic nervous system (SNS), and endothelin overproduction [32].

Early evaluation of renal function in acute HF is essential for optimizing diuretic therapy [33, 34]. Detecting worsening renal function, indicated by a rise in serum creatinine > 0.3 mg/dL or a drop in eGFR of 25%, guides prognosis. True worsening renal function necessitates pharmacological optimization and possibly renal replacement therapy, whereas pseudo-worsening renal function suggests neurohormonal blockade without a worse prognosis [33].

HF and chronic kidney disease (CKD) are intertwined, exacerbating each other through shared pathogenic mechanisms, collectively referred to as CRS [35]. CKD predisposes to HF *via* fluid retention, while HF can induce CKD by reducing renal perfusion [35, 36]. New biomarkers, such as cardiac troponin and NT-proBNP, are being employed for early diagnosis and monitoring of CRS [37].

Neurohormonal pathways intricately regulate cardiac and renal functions. Enhanced RAAS activation and sympathetic activity contribute to systemic vasoconstriction and fluid retention [38]. These compensatory mechanisms lead to fibrogenesis in both the heart and kidneys, ultimately contributing to multi-organ dysfunction.

Neurohormonal activation can exacerbate CRS through the release of inflammatory interferons and hormones that induce oxidative stress and inflammation, causing further kidney damage [38]. The imbalance between vasoconstrictive and vasodilatory factors triggers pathologic volume expansion and multi-organ fibrosis, accelerating HF progression [38].

### 3.4 Respiratory Comorbidities

COPD and Obstructive Sleep Apnea (OSA) are noteworthy non-cardiovascular comorbidities in HF, with approximately one-third of HF patients presenting with coexisting COPD 39-42 [39-42]. The prevalence of COPD is 16% in heart failure with HFrEF and 14% in HFpEF [42].

The concomitant presentation of COPD and HF complicates diagnosis due to overlapping symptoms, underscoring the importance of diligent screening for COPD among HF patients [40]. These patients experience an unfavorable clinical course, with a higher incidence of additional comorbidities like hypertension (60-80%), peripheral vascular disease (20-30%), diabetes, and atrial fibrillation (20-40%). Although 75% of HF deaths are cardiovascular in nature, COPD coexistence heightens the adjusted risk of non-cardiovascular mortality, such as malignancy, pneumonia, and respiratory failure [40]. Moreover, one study posits that non-cardiovascular comorbidities like COPD exert a more detrimental impact on HFpEF than on HFrEF [40].

COPD increases the risk of both morbidity and mortality in chronic and acute HF [41]. While a European HF survey associated chronic kidney disease, anemia, and diabetes with higher all-cause mortality in HF patients, COPD's significance was borderline [39]. However, a meta-analysis did find that COPD significantly elevates long-term all-cause mortality, especially among HFrEF patients, though not due to cardiovascular causes [43]. Data from the multicenter, double-blind, randomized GISSI-HF trial also identified COPD as an independent predictor of increased mortality, elevating the risk by 28% [40].

COPD and HF share several etiological factors, including smoking, age, and systemic inflammation, which cumulatively impact exercise tolerance and quality of life [44, 45]. In the pathophysiological landscape, low-grade systemic inflammation promotes airway and alveolar remodeling in COPD and contributes to the development of ischemic heart disease and HF [46].

Pulmonary hypertension in COPD, resulting from chronic hypoxemia and inflammation, may lead to right ventricular hypertrophy and eventually right-sided HF, also known as cor pulmonale [44]. Furthermore, increased pulmonary vascular resistance due to inflammatory reactions can exacerbate pulmonary hypertension and contribute to HF development [44].

Regarding therapeutic approaches, bronchodilators improve respiratory symptoms and cardiac output while reducing pulmonary hyperinflation, thus preventing acute COPD exacerbations and cardiovascular events [42-50]. Yet, no formal interaction between COPD and Left Ventricular Ejection Fraction (LVEF) has been established. Routine spirometry screening for COPD among HF patients is recommended to facilitate early and effective treatment.

### 3.5 Sleep-related Breathing Disorders (SBD)

SBDs, predominantly OSA and Central Sleep Apnea (CSA), are common in HF patients and contribute to poor outcomes [41]. The prevalence of CSA varies from 30-60%, with underreporting and inadequate screening likely inflating the higher end of that range [39-47]. Both OSA and CSA are frequently observed in HF patients-approximately three out of ten in HFrEF and four out of ten in HFpEF [42]. Several risk factors, including higher functional class according to the NYHA, waking hypocapnia, and lower ejection fraction, are associated with CSA [47]. Conversely, conventional risk factors like Body Mass Index (BMI), age, and gender are not reliable predictors for OSA, necessitating more frequent use of polysomnography [41].

Untreated OSA is associated with increased mortality in HFrEF, independent of confounding variables, while CSA is a recognized independent risk factor for adverse HF outcomes [42-47]. Both conditions are linked with increased hospital readmissions in HF patients [36-42]. Despite these associations, current therapeutic interventions such as Adaptive Servo-Ventilation (ASV) do not improve cardiovascular prognosis and may even worsen it [41, 42]. Therefore, routine screening for SBD in HF patients is essential.

### 3.6 Obesity

Obesity increases the risk of Heart failure so much so that for every 5 kg/m^2^ increase in BMI, the risk of HF increases by 41% [48]. Recent studies have suggested that higher BMI is more strongly associated with HFpEF than HFrEF [49].

Several direct and indirect mechanisms have been proposed to explain the impact of high BMI on the development of HFpEF. A higher amount of visceral adipose tissue is associated with abnormalities in cardiac structure and function, as well as plasma volume expansion and impairment in LV relaxation through systemic inflammation, thereby causing cardiac fibrosis and HFpEF [50-52]. Indirectly, obesity contributes to the burden of hypertension, diabetes mellitus and coronary artery disease, which are known traditional risk factors for HF [49].

While obesity remains a greater risk factor for the development of HF, some studies have demonstrated a phenomenon of obesity paradox (mechanism unknown), whereby obese patients with HFpEF have lower mortality risk as compared with underweight (*i.e*., BMI <18.5 kg/m^2^) and normal-weight (*i.e*., BMI 18.5-24.9 kg/m^2^) patients [49, 53, 54].

Interestingly, in spite of the well-recognized obesity paradox, there is clinical evidence that intentional weight loss, including bariatric surgery, is beneficial in treating HF patients with obesity [55, 56]. Exercise training has also demonstrated improvement in the quality of life of both HFpEF and HFrEF patients [57]. However, the effects of long-term weight loss strategies on clinical outcomes remain unknown [57].

### 3.7 Thyroid Disorders

In HF, cardiac injury triggers changes in the thyroid hormone signaling, encouraging compensatory structural remodeling of the heart *via* cardiomyocyte proliferation, resulting in cell hypertrophy, which soon turns maladaptive [58].

The two hypothyroid states most commonly investigated in HF settings are subclinical hypothyroidism (SCH) and low triiodothyronine syndrome (LT3S). LT3S has been suggested to be a marker of chronic illness as its incidence is higher in HF patients than in those with acute coronary syndrome [59]. Both LT3S and SCH have also been associated with markers of clinical severity in both patients with HFrEf and HFpEF [58, 60]. In patients with HF, low T3 levels are linked to higher cardiac and all-cause mortality [61, 62].

Thus, it is important to consider the role of thyroid abnormalities during the prognostic stratification of HF patients. There is still clinical discrepancy regarding which form of thyroid hormone (T3 or T4) and which method of administration (oral *vs.* I.v.) is more beneficial but this much is obvious that pharmacological hyperthyroidism must not occur [60, 63, 64].

### 3.8 Hyperkalemia

Hyperkalemia is common in HF patients and undermines treatment efficacy, often leading to hospitalization [65]. The 2022 Diamond trial involving 1,642 patients found that hyperkalemia increases mortality and arrhythmia risk in HF as summarized in Fig. (**2**) [66]. This complicates the use of RAAS inhibitors, which are effective for HF but often cause hyperkalemia. Managing this condition becomes particularly challenging due to confounding risk factors like diabetes, advanced age, and chronic renal failure [67]. Consequently, meticulous monitoring of potassium levels and renal function is critical, especially when RAAS inhibitors are prescribed [68, 69].

### 3.9 Cognitive Dysfunction

Cognitive dysfunction, affecting 43% of HF patients, is increasingly recognized for its impact on treatment planning and medication adherence [69]. While the underlying mechanisms are not fully understood, factors like reduced cerebral blood flow and inflammation are implicated. Depression, atrial fibrillation, and other conditions further contribute to this dysfunction [70]. Neuroimaging supports the early onset of cognitive deficits, even with mild left ventricular dysfunction. Effective interventions include comprehensive care, educational programs, and cognitive training [71].

### 3.10 Hypertension and HF

Hypertension and HF are intricately linked, each exacerbating the other's progression. Hypertension is not only a significant precursor to HF but also a risk factor for other cardiovascular diseases like stroke and coronary artery disease [72]. Mechanistically, chronic pressure overload from hypertension leads to left ventricular hypertrophy (LVH), affecting both HFpEF and HFrEF patients [73, 74]. Over time, this can evolve into diastolic dysfunction, a precursor to symptomatic HF, characterized by impaired ventricular filling and elevated left atrial pressure [72]. Among long-standing hypertensives, a subset progresses to HFrEF, often due to a disproportionate loss of myocytes, leading to dilated cardiomyopathy Table **1** [75, 76].

### 3.11 Autonomic Dysfunction in HF

The main characteristic of the HFpEF and HFmrEF is the reduced diastolic function, causing elevation of the filling pressure and reducing the compliance of the LV [77]. The part of the neurohormonal activation, specifically the autonomic disbalance, is critical in the pathophysiology of these conditions, where the sympathetic nervous system has higher activity while the parasympathetic nervous system is depressed, displayed as a low cardiac vagal tone [77, 78] which increases the heart rate, promotes the retention of sodium and water, and reduces the heart rate variability (HRV) [77]. These events encouraged the researchers to investigate the application of regulatory intervention on the autonomic system. The outcomes of the utilization of transcutaneous vagus nerve stimulation in a population with HFpEF suggest that the increased vagal tone reaches the autonomic system balance, leading to a continued improvement in LV ejection fraction and the heart rate recovery (HRR), referring to the rate at which the HR decreases in the recovery form exercise [78]. Even though, after a 12-month therapy with vagal stimulation therapy, the population with HFpEF and HFmrEF did not show effects on the mechanical measurement (filling pressure marker and LV mass index), the clinical improvement was evident in NYHA Class, tolerance of walk distance for 6 minutes and the life quality with a low adverse events rate [77].

## 4 EVIDENCE-BASED MANAGEMENT STRATEGIES FOR HF AND COMORBIDITIES

### 4.1 Pharmacological Approaches for Heterogeneous HF Phenotypes

HF patients are categorized based on LVEF into HFpEF (LVEF ≥ 50%), HFmrEF (LVEF 41-49%), and HFrEF (LVEF < 40%) [6]. Despite stabilization in overall HF incidence due to advances in cardiovascular care, HFpEF cases are rising, partly due to the lack of targeted therapies [6, 79]. This emphasizes the need for phenotype-specific treatments [79].

The 2021 ESC guidelines recommend an array of medications for managing HFrEF, such as angiotensin-converting enzyme (ACE) inhibitors, Angiotensin receptor-neprilysin inhibitors (ARNIs), beta-blockers, and Mineralocorticoid receptor antagonists (MRAs) [6]. The 2023 ESC task force update has recommendations for the diagnosis and treatment of heart failure (HF), building on the 2021 ESC HF guidelines. Notably, SGLT2 inhibitors now receive a class IA recommendation for treating HFmrEF and HFpEF. The use of hydrochlorothiazide and acetazolamide alongside loop diuretics enhances decongestion in acute HF, though it doesn't alleviate symptoms or prevent rehospitalizations. Commencing and adjusting chronic HF therapies during hospitalization for acute HF is linked to better outcomes. Increasing evidence supports the positive impact of IV iron in HFrEF patients, improving symptoms and exercise capacity, and potentially modestly reducing HF hospitalizations [80]. Moreover, emerging non-steroidal MRAs like Finerenone show kidney-sparing effects [81]. ARNIs have proven superior to traditional ACE inhibitors, such as Enalapril, in improving outcomes [82]. A 2022 meta-analysis supports the synergistic benefits of combining ARNIs, beta-blockers, MRAs, and SGLT2 inhibitors, as mentioned in Table **2** [83]. However, combining Aliskiren and Enalapril is cautioned against, especially in diabetics, due to adverse effects without added benefits [84].

Treatments for HFpEF remain suboptimal, complicated by prevalent comorbidities like obesity, hypertension, and diabetes [85, 86]. Trials for Spironolactone and Sacubitril-Valsartan showed no marked improvement in outcomes [87-89]. In an emperor preserved trials, it was found that regardless of whether diabetes was present or not, empagliflozin decreased the combined risk of cardiovascular mortality or heart failure hospitalization in individuals with heart failure and a maintained ejection fraction [90].

Contrastingly, A 2021 study on diuretic efficacy highlighted the absence of a significant difference in key outcomes, emphasizing the need for more research [91].

As with HF with decreased EF (HFrEF) or HF with preserved EF (HFpEF), HFmrEF has clinical characteristics with other HF phenotypes, such as a high prevalence of ischemic etiology and benefits from the cornerstone medications advised for HFrEF. Of all the HF phenotypes, HFmrEF has the highest incidence of transition to the severe systolic dysfunction profile, that is the goal of disease-modifying treatments, and it also has the worst prognostic consequences [92]. For HFmrEF, therapeutic strategies are adapted from HFrEF in the absence of targeted research. Preliminary data suggest that traditional HFrEF medications may offer some benefit [6].

In summary, the landscape of HF management is gradually becoming more phenotype-specific, but gaps persist. Treatments for HFrEF are established and effective, whereas those for HFpEF and HFmrEF require further exploration. The field is on the verge of a more nuanced pharmacotherapeutic approach, necessitating ongoing research for efficacious treatment regimens tailored to each HF subtype.

### 4.2 Management of Anemia in HF: Role of Iron Supplementation

Anemia and iron deficiency are common comorbidities in HF that influence clinical outcomes [93]. Iron deficiency in HF is bifurcated into absolute and functional types, affecting 15% and 18% of patients, respectively [94, 95]. Intravenous (I.V.) iron therapy improves exercise capacity and reduces hospitalizations, albeit without altering mortality rates [96]. Conversely, the IRONOUT-HF study found no benefit for oral iron therapy in HFrEF patients [97]. The study was, however, limited by its narrow focus and statistical power [97]. I.V. iron is favored due to increased hepcidin levels, showing long-term benefits like improved symptoms and reduced hospitalizations [95, 98]. Personalized iron dosages, often calculated using the Ganzoni formula, are crucial to prevent overload and potential coronary events [94, 99].

### 4.3 Management of DM in HF

Selecting the right medication is essential for HF patients with concurrent diabetes. SGLT2 inhibitors are recommended per 2022 guidelines [9]. Meta-analyses show these inhibitors significantly reduce mortality and hospitalizations in both HFrEF and HFpEF patients, also improving Kansas City Cardiomyopathy Questionnaire (KCCQ) scores [100, 101].

The DELIVER trial aimed to explore the benefits of administering 10 mg oral dapagliflozin in HF patients with mildly reduced or preserved ejection fraction > 40% [102]. The trial found dapagliflozin to be effective across all glycemic subgroups.

The PRESERVED-HF trial specifically demonstrated that dapagliflozin use substantially improved the quality of life in HFpEF patients [103]. This improvement was noteworthy, especially in comparison with other trials that used empagliflozin under similar conditions [104, 105].

In addition to the cardiovascular benefits, cardiovascular outcome trials (CVOTs) like EMPA-REG and CREDENCE demonstrated the long-term nephroprotective effects of SGLT2 inhibitors [106]. This included a reduction in albuminuria by 30%, along with a decrease in the risk of progression to ESRD by 30-40% [106]. Although the use of SGLT2 inhibitors lowers the eGFR initially by about 5 ml/min/1.73m^2^, the value gradually reaches the pretreatment values over a period of 3-9 months [107]. Data from CVOTs like EMPA-REG, CREDENCE, and DAPA-CKD show that patients treated with SGLT2 inhibitors had a slower eGFR decline as compared to patients treated with placebo [107].

#### 4.3.1 Comparative Efficacy of Antidiabetic Agents

A meta-analysis of 23 cardiovascular outcome trials comparing the effectiveness of glucagon-like peptide-1 receptor agonists (GLP-1RA), SGLT2 inhibitors, and dipeptidyl peptidase-4 (DPP-4) inhibitors showed a superior reduction in HF hospitalizations and mortality by SGLT2 [108]. Notably, there were no trials directly comparing these classes, limiting the conclusions.

#### 4.3.2 Role of Metformin

Metformin, despite its theoretical contraindication in HF due to a risk of lactic acidosis, remains widely used [109, 110]. Recent evidence suggests that the risk of lactic acidosis is attributable to comorbidities than to metformin itself [110-112]. Combination therapy with metformin and either GLP-1RA or SGLT2 inhibitors has shown efficacy in both glycemic control and cardiovascular benefits [113].

#### 4.3.3 Considerations for Tailored Therapies

Both European and American guidelines align in advocating for personalized diabetic HF treatments [114]. Treatment adjustments in HFrEF are generally guided more by comorbidities than by ejection fraction, incorporating medications like ACE inhibitors and ARBs based on clinical criteria [108]. In HFpEF, the focus remains on symptom control, as existing treatments haven’t significantly impacted mortality or morbidity [114].

### 4.4 Management of COPD in HF

For individuals with both HF and COPD, an integrated approach to treatment is essential. Core therapeutic agents for HF include beta-blockers, ACE inhibitors or ARBs, diuretics, and statins. Notably, selective Beta1-blockers such as Metoprolol, Nebivolol, and Bisoprolol are recommended for COPD patients with HF to maintain lung function, with a preference for Bisoprolol. These beta-blockers are particularly important during acute exacerbations to reduce mortality and recurrence rates. Loop diuretics should be administered cautiously in this population to prevent complications such as metabolic alkalosis, hypoventilation, and hypercapnia [44].

Aldosterone antagonists like spironolactone improve survival across the board in HF patients, including those with COPD. Meanwhile, inhaled beta-agonists, when combined with selective Beta1-blockers, remain safe for COPD patients. Non-selective beta-blockers should be avoided in this patient cohort. Beta1-blockers also mitigate the cardiac risks associated with Beta2-agonist usage. ACEIs or ARBs may alleviate pulmonary airway constriction and enhance outcomes related to HF symptoms, hospitalization rates, and mortality. Antimuscarinic long-acting inhaled bronchodilators are preferred over Beta2-agonists for combined COPD and HF management. Corticosteroids should be used judiciously in HF patients to minimize the risk of fluid retention [44].

### 4.5 Management of Sleep Apnea in HF

The European Society of Cardiology advises comprehensive screening for HF patients, including evaluations for sleep apnea. If OSA is diagnosed, continuous positive airway pressure (CPAP) is the recommended treatment [115, 116]. Previous research has indicated that CPAP therapy significantly improves cardiac function and may even reverse cardiac remodelling when combined with standard medications [117]. CPAP therapy not only improves sleep quality but also enhances left ventricular ejection fraction within three months of treatment initiation [118]. Furthermore, CPAP therapy has been associated with reduced mortality rates and improved quality of life [119]. There are also benefits in terms of myocardial sympathetic nerve function and overall cardiac efficiency, particularly in patients with severe OSA [120].

However, patient adherence to CPAP remains suboptimal, with 8-15% discontinuing treatment as early as the first night [121, 122]. This highlights the need for additional research to identify specific patient populations that would benefit most from CPAP and to develop alternative treatment methods. Preliminary studies have suggested that exercise may be a viable alternative to CPAP for managing OSA in HF patients (Table **2**), leading to improvements in daytime sleepiness, quality of life, peak oxygen consumption, and muscle performance [123]. Future research is essential for validating these findings and establishing the efficacy of various treatment options for sleep-related disorders co-existing with HF.

## 5 CHALLENGES IN HF RESEARCH AND CLINICAL PRACTICE

Classification of HF, crucial for tailored treatment strategies, is inconsistent and poses significant challenges. The NYHA, for instance, classifies HF based on symptom severity rather than underlying pathology, resulting in heterogeneous patient populations in trials and affecting the generalizability of research outcomes [6, 124-126]. Moreover, the research pace, particularly in RCTs, is slowed by ethical, regulatory, and financial hurdles, as well as patient recruitment issues [111, 127-129].

Treatment complexity, often involving polypharmacy, heightens the risk of adverse interactions, as seen with Sacubitril/Valsartan and its interactions with Furosemide and Atorvastatin [130-136]. Patient non-compliance further complicates management due in part to social and logistical challenges [131, 137].

Despite these issues, non-pharmacological interventions like exercise therapy have shown promise in treating HF-related complications such as sleep apnea [123]. Addressing these complexities requires a standardized approach to treatment, perhaps utilizing methods like Latent Class Analysis (LCA) to target patient better needs (Table **3**) [138-140].

## CONCLUSION

The high incidence of comorbidities in HF patients significantly affects clinical outcomes and necessitates an integrated approach to care. This review highlights the impact of comorbid conditions such as anemia, DM, and RI on HF management and mortality. Robust screening strategies are urgently needed to identify these conditions, some of which may be underreported or present overlapping symptoms.

Pharmacological treatments are effective but also come with their own set of risks, such as hyperkalemia, requiring a balanced clinical judgment. Patient compliance adds another layer of complexity, more so among those with cognitive impairments. Non-pharmacological approaches, like exercise therapy, also offer effective management options for complications like sleep apnea.

Despite extensive research, significant gaps remain in understanding optimal treatment for HF with comorbidities. These gaps underline the need for RCTs, though their design and implementation are fraught with challenges including ethical considerations and funding constraints. Future treatment strategies for HF must hinge on refining our understanding of its complex interplay with comorbidities and translating this knowledge into personalized care plans.

## Figures and Tables

**Fig. (2):**
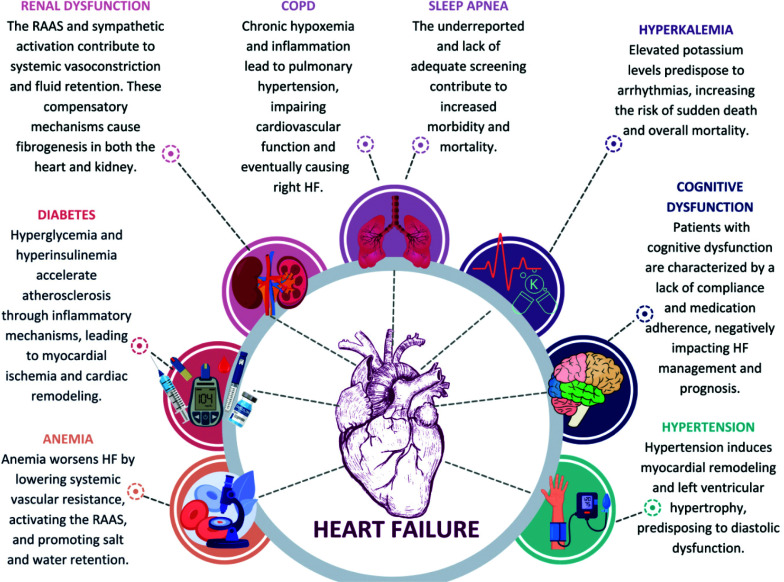
Impact of comorbidities on prognosis of HF patients. **Abbreviations:** COPD = Chronic Obstructive Pulmonary Disease; HF= Heart Failure; RAAS= Renin-angiotensin-aldosterone system. **Source:** Created using Canva (canva.com). Image template creators are acknowledged in the acknowledgements section.

**Table 1:** Prevalence and impact of comorbidities in Heart Failure.

**Study Title**	**Study Design**	**Sample Size**	**Comorbid Conditions Studied**	**Results and Limitations**
Trends in noncardiovascular comorbidities among patients hospitalized for heart failure: insights from the get with the guidelines-heart failure registry [7]	Retrospective analysis	207984 patients	HF	Based on data from Medicare, 40% of patients in the program suffer from HF also have at least five non-cardiovascular conditions. The patients are voluntary beneficiaries, meaning that the studied population may not be a true representation of all HF patients in the US.
Prevalence of, associations with, and prognostic role of anemia in heart failure across the ejection fraction spectrum [13]	Observational Study	49985 patients	Anemia	Anemia was diagnosed in 34% of the sample, being more prevalent in the group with HFpEF.
Anemia and its association with clinical outcome in heart failure patients undergoing cardiac resynchronization therapy [16]	Observational study	300 patients	Anemia	Cardiac resynchronization therapy patients presented a worst prognosis when they were diagnosed with persistent anemia.
Association between anemia and outcome in patients hospitalized for acute heart failure syndromes: Findings from Beijing Acute Heart Failure Registry (Beijing AHF Registry) [17]	Retrospective study	3279 patients	Anemia	HF associated with anemia signified a mortality rate of 38,4%. The study did not consider the identification of iron deficiency.
Improved and new-onset anemia during follow-up in patients with acute decompensated heart failure [18]	Observational study	771 patients	Anemia	Patients recovering from acute decompensated HF who presented persistent or recently diagnosed anemia showed a higher risk of all-cause death and increased hospitalization rate.
Heart failure in people with type 2 diabetes vs. those without diabetes: A retrospective observational study from South India [20]	Retrospective observational study	397 patients	DM	1/3 of patients with HF also have DM. The prevalence of hypertension and coronary artery disease was higher in the group with type 2 diabetes and heart failure compared to the group without HF.
Diabetes and pre‐diabetes in patients with heart failure and preserved ejection fraction [21]	Pos-hoc analysis of RCT	4796 patients	DM and pre-diabetes	The prevalence of DM in the patient population was 38%, while the prevalence of pre-diabetes was 28%
Diabetic phenotype and prognosis of patients with heart failure and preserved ejection fraction in a real-life cohort [22]	Prospective cohort study	183 patients	DM	It was identified that several characteristics are shared by patients having concomitant diagnoses of HFpEF and DM. Among the small sample of a single center, they tended to be younger and obese.
Diabetes, preclinical heart failure stages, and progression to overt Heart Failure: The atherosclerosis risk in communities (ARIC) Study [23]	Prospective cohort study	4774 patients	DM	Patients with uncontrolled DM (HbA1c ≥7%) have a higher risk of developing heart failure in stages A and B. The risk is greater in stage B than in stage A. The study only applied to older individuals, so younger adults may not experience the same results.
Diabetes mellitus and the causes of hospitalization in people with heart failure [24]	Prospective cohort study	711 patients	DM	Cause-specific analyses showed that people with DM had a significantly higher burden of hospitalization due to decompensated heart failure, other cardiovascular events, and infections, but not other non-cardiovascular events. The study did not investigate other factors that could increase hospitalization duration
Impact of diabetes mellitus on mortality in patients with acute heart failure: A prospective cohort study [25]	Prospective cohort study	5625 patients	DM	DM was associated with higher mortality risk in acute heart failure, especially in patients with reduced ejection fraction. Well-managed diabetes (HbA1c < 7.0%) is linked to lower mortality risk than uncontrolled diabetes.
Impact of baseline renal dysfunction on cardiac outcomes and end-stage renal disease in heart failure patients with mitral regurgitation: The COAPT trial [28]	RCT	614 patients	RD	Baseline RD was prevalent in HF patients with severe Mitral Regurgitation. Interventions targeting the mitral valve using TMVr techniques can positively impact both cardiac and renal outcomes. It could not be determined how frequently baseline RD represented stable chronic kidney disease or whether any comorbidities were enrolled in the RCT.
The association between changes in echocardiography and risk of heart failure hospitalizations and death in adults with chronic kidney disease [29]	Prospective multicenter cohort Study	2673 patients	CKD	Patients with CKD exhibited greater LV structural alteration and decreased LV ejection fraction. These patients had a higher risk of hospitalization and mortality.
The impact of kidney dysfunction categorized by urinary to serum creatinine ratio on clinical outcomes in patients with heart failure [30]	Observational study	1009 patients	RD	Based on UC/SC ratio analysis, the prevalence rates of intrinsic, intermediate, and pre-renal KD were 23%, 30%, and 47% respectively. The group with intrinsic KD had the highest cardio-renal event rate compared to the other groups. In this investigation, the fractional excretion of sodium and urea was not assessed, so it is unclear whether the UC/SC ratio was suitable for categorizing kidney dysfunction in patients with heart failure.
Impact of renal impairment on beta-blocker efficacy in patients with heart failure [31]	RCT	16740 patients	RD	eGFR is independently associated with mortality, with a 12% higher risk of death for every 10 ml/min/1.73 m^2^ lower eGFR. There were insufficient patients with severe renal dysfunction (eGFR <30 ml/min/1.73 m^2^) to draw conclusions.
Co-morbidities in patients with heart failure: an analysis of the European Heart Failure Pilot Survey [39]	Observational study	3226 patients	CKD, anemia, diabetes, stroke, COPD, sleep apnea, hyper and hypothyroidism.	In the studied population, HF was diagnosed by the treating doctor. The analysis indicated that comorbidities represented an independent risk factor for mortality and hospitalization.
Prevalence and Prognostic Impact of Chronic Obstructive Pulmonary Disease in Patients with Chronic Heart Failure: Data from the GISSI-HF Trial [40]	RCT	6975 patients	COPD	COPD is an independent predictor of mortality and hospitalization in ambulatory HF patients. Increased awareness and improved management of COPD may significantly reduce the burden of this morbidity to patients with HF. However, the evaluation of the prognostic impact of COPD in HF patients was not the primary end point of this study.
The impact of chronic obstructive pulmonary disease on hospitalization and mortality in patients with heart failure [43]	Meta-analysis	18 studies	COPD	COPD as a comorbidity could exacerbate the risk of all-cause mortality of the HF patients. However, there are relatively fewer studies with some wide Cl’s. Also, there is significantly low findings on the effects on hospitalization
Patiromer for the management of hyperkalemia in heart failure with reduced ejection fraction: The DIAMOND trial [66]	RCT	1642 patients	Patiromer, and MRAs/ Hyperkalemia	Hyperkalemia occurrence was reduced using patiromer concurrently and MRAs. However, the investigation was limited by factors induced by the pandemic secondary to COVID-19.
Cognitive impairment and heart failure: systematic review and meta-analysis [70]	Meta analysis	37 studies were eligible with 8411 participants	Cognitive Dysfunction	The presence of cognitive dysfunction in 43% of the cases of HF implied a lack of adherence to the treatment, complicating the medical plaining. Additional investigation is necessary.
Prehypertension is associated with abnormalities of cardiac structure and function in the atherosclerosis risk in communities study [73]	Observational study	4871 patients	Hypertension	The group diagnosed with prehypertension had high rates of cardiac morphology alteration but not as much as the patients with frank hypertension. Diastolic dysfunction in mild and moderate to severe grade was observed in individuals with prehypertension.

**Table 2:** Pharmacological therapies and evidence-based management strategies for HF and comorbidities.

**Study**	**Study Design**	**Sample Size**	**Comorbid Conditions or Therapy studied**	**Results and Limitations**
Angiotensin-neprilysin inhibition *versus* enalapril in heart failure [82]	RCT	8442 patients	Angiotensin receptor-neprilysin inhibitor LCZ696 or Enalapril.	Lower rates of death and hospitalization for HF were seen in patients using LCZ696 compared to those treated with enalapril. To reach the projected rates, the researchers recruited individuals with elevated natriuretic peptide.
A systematic review and network meta-analysis of pharmacological treatment of heart failure with reduced ejection fraction [83]	Meta-Analysis	75 trials/ 95.444 patients	Pharmacological therapy for HFrEF	The study concluded that beta-blockers (BB), angiotensin receptor-neprilysin inhibitors (ARNi), mineralocorticoid receptor antagonists (MRAs), and sodium-glucose cotransporter-2 inhibitors (SGLT2i), together had the lower rate of deaths for any cause, cardiovascular deaths, and hospitalization for the first time secondary to HF.
Aliskiren, enalapril, or aliskiren and enalapril in heart failure [84]	RCT	8835 patients	Aliskiren, Enalapril, or Aliskiren and Enalapril.	Associating aliskiren with enalapril was inefficient in treating chronic HF; it also caused higher rates of adverse events.
Spironolactone in patients with heart failure, preserved ejection fraction, and worsening renal function [87]	RCT	1767 patients	Worsening Renal Failure (WRF)/ spironolactone treatment.	WRF occurred in more elevated rates in patients treated with spironolactone compared. Nonetheless, when using spironolactone, the events of cardiovascular deaths were lower, even in patients with WRF.
Angiotensin-neprilysin inhibition in heart failure with preserved ejection fraction [88]	RCT	4822 patients	Angiotensin-Neprilysin Inhibition.	Participants with HFpEF treated with Sacubitril-valsartan did not present a reduction in hospitalizations or mortality for cardiovascular events. It must be taken into consideration that the study did not include patients with high risk and those incapable of adherence to pharmacological treatment.
Empagliflozin empagliflozin in heart failure with a preserved ejection fraction [90]	Meta-Analysis	23492 patients	Beta-Blockers (BB); inhibitors of RAAS.	BBs may decrease the risk of death from cardiovascular causes, but there is no certainty. The tendency to have lower hospitalization rates was seen in patients using MRAs, while ACEIs and ARBS have no significant protective effects.
Comparative effects of furosemide and other diuretics in the treatment of heart failure: A systematic review and combined meta-analysis of randomized controlled trials [91]	Systematic review and combined meta-analysis of randomized controlled trials (RCTs)	54 studies and 10.740 patients	Diuretics in HF	Diuretics, such as azosemide and torasemide, decrease brain natriuretic peptide (BNP) levels. Treatment with torasemide showed effectiveness in reducing collagen volume fraction and edema. The paper included a limited number of studies.
Absolute and functional iron deficiency is a common finding in patients with heart failure and after heart transplantation [95]	Clinical trial	399 patients	ID	In the group of HF patients, absolute iron deficiency and functional iron deficiency were prevalent in 15% and 18%, respectively. In the group of heart transplant recipients, absolute iron deficiency occurred in 30% of patients, whereas functional iron deficiency was detected in 17% of cases.
Effects of intravenous iron therapy in iron-deficient patients with systolic heart failure: a meta-analysis of randomized controlled trials [96]	Meta-analysis	851 patients	ID	Patients with systolic HF associated with iron deficiency evolve with better prognosis, exercise tolerance and quality of life, and improvement of HF symptoms after receiving IV iron therapy.
Effect of oral iron repletion on exercise capacity in patients with heart failure with reduced ejection fraction and iron deficiency: The ironout HF randomized clinical trial [97]	RCT	225 patients	ID	HF patients with iron deficiency presented no benefits after being treated with oral iron.
Comparative efficacy of intravenous and oral iron supplements for the treatment of iron deficiency in patients with heart failure: A network meta-analysis of randomized controlled trials [98]	Meta Analysis	5205 patients	ID	Positive outcomes of treating iron deficiency in HF patients, such as improvement of exercise tolerance and quality of life, resulted after treatment with intravenous iron. On the other hand, management with oral iron did not show significant benefits; however, further research on oral iron is needed for a more certain conclusion.
SGLT2 inhibitors in patients with heart failure with reduced ejection fraction: A meta-analysis of the EMPEROR-Reduced and DAPA-HF trials [100]	Meta-Analysis	8474 patients	SGLT2 inhibitors in patients with HFrEF	These agents improved renal outcomes and reduced all-cause and cardiovascular death in patients with HFrEF.
Efficacy and safety of dapagliflozin in patients with heart failure with mildly reduced or preserved ejection fraction by baseline glycemic status (DELIVER): A subgroup analysis from an international, multicenter, double-blind, randomized, placebo-controlled trial [102]	RCT	6263 patients	Dapagliflozin/ Patients with HFmrEF and HFpEF	Dapagliflozin improved HF outcomes in individuals with both slightly reduced and preserved ejection fraction.
The SGLT2 inhibitor dapagliflozin in heart failure with preserved ejection fraction: A multicenter randomized trial [103]	RCT	324 patients	The SGLT2 inhibitor dapagliflozin	Dapagliflozin significantly decreased the risk of worsening heart failure or death independently of the diabetic status. Part of analysis was post-hoc, only sub-group analysis.
Cardiovascular and renal outcomes with empagliflozin in heart failure [104]	RCT	3730 patients	Empagliflozin in HF	Empagliflozin group had a lower risk of cardiovascular death or hospitalization for heart failure than those in the placebo group.
Dapagliflozin in patients with heart failure and reduced ejection fraction [105]	Phase 3, placebo-controlled trial.	4744 patients	Dapagliflozin in HF	Compared to placebo, patients with HF using dapagliflozin (an SGLT2 inhibitor) reduced the risk of worsening heart failure or death from cardiovascular causes.
The effect of DPP-4 inhibitors, GLP-1 receptor agonists and SGLT-2 inhibitors on cardiorenal outcomes: A network meta-analysis of 23 CVOTs [108]	Meta-Analysis	181143 patients	DPP-4 inhibitors, GLP-1 receptor agonists and SGLT-2 inhibitors	SGLT-2 inhibitors and GLP-1RA are superior to DPP-4 inhibitors in reducing the risk of most cardiorenal outcomes; SGLT-2 inhibitors are superior to GLP-1RA in reducing the risk of HHF and renal events.
Meta-analysis comparing outcomes of therapies for patients with central sleep apnea and heart failure with reduced ejection fraction [118]	Meta-Analysis	19 RCTs	OSA/CPAP therapy	CPAP demonstrated positive short-term (3 months) outcomes on quality of life and sleep in patients with HF and Sleep Apnea. However, there was no long-term cardiovascular benefit, decrease in mortality, or improvement in overall quality of life.
Effects of short-term continuous positive airway pressure on myocardial sympathetic nerve function and energetics in patients with heart failure and obstructive sleep apnea: a randomized study [120]	RCT	45 patients	OSA/CPAP therapy	Short-term CPAP improved myocardial sympathetic nerve function in patients with HFrEF and OSA, with no improvement in overall energetics.
One-year adherence to continuous positive airway pressure with telemonitoring in sleep apnea hypopnea syndrome: A randomized controlled Trial [121]	RCT	120 patients	OSA/CPAP	Monitoring patients with OSA who are managed with CPAP *via* telemedicine *vs*. usual care did not significantly differ in the therapy’s adherence. However, telemedicine could reduce the need for visits in person.
Effects of exercise training and CPAP in patients with heart failure and OSA: A preliminary study [123]	RCT	65 participants	OSA/CPAP therapy	CPAP improves the functional capacity of the heart in patients with Congestive Heart Failure. Yet, the study demonstrated that exercise improved the quality of life more than CPAP. Both CPAP and exercise together improved the quality of life even more.

**Table 3:** Challenges and limitations in heart failure research and clinical practice.

**Study**	**Study Design**	**Sample Size**	**Comorbid Conditions or Therapy Studied**	**Results and Limitations**
Angiotensin-neprilysin inhibition in heart failure with preserved ejection fraction [88]	RCT	4822 patients	Angiotensin-Neprilysin Inhibition.	Participants with HFpEF treated with Sacubitril-valsartan did not present a reduction in hospitalizations or mortality for cardiovascular events. It must be taken into consideration that the study did not include patients with high risk and those incapable of adherence to pharmacological treatment.
Effect of the angiotensin receptor-neprilysin inhibitor sacubitril/valsartan on the pharmacokinetics and pharmacodynamics of a single dose of furosemide [133]	Clinical Trial	28 patients	Sacubitril/Valsartan	When using Sacubitril/valsartan, furosemide’s bioavailability and 24-hour urinary excretion did not modify significantly. However, the sample was small, considering the limitations of the study.
‘Patient Lost to Follow-up’: Opportunities and Challenges in Delivering Primary Care in Academic Medical Centers [137]	Retrospective cohort study	Visits from 7/1/2014 to 6/30/2019; 89 physicians (51%) participated in a qualitative analysis.	Chronic illness burden	Effective pharmacological adjustment and the early detection of signs of adverse drug reactions are possible in patients who fully adhere to their treatment and maintain follow-up.
Type 2 diabetes and heart failure: Characteristics and prognosis in preserved, mid-range and reduced ventricular function [138]	Observational study	30696 patients	Type 2 Diabetes	T2DM is an independent mortality predictor across all HF entities increasing mortality risk by 30%-50%.
Model-based comorbidity clusters in patients with heart failure: Association with clinical outcomes and healthcare utilization [140]	Regression analysis	318384 patients	Comorbidities burden	It is necessary to adjust the management of HF, considering comorbidities of each patient.

## LIST OF ABBREVIATIONS

COPD: Chronic Obstructive Pulmonary Disease
DM: Diabetes Mellitus
EF: Ejection Fraction
HF: Heart Failure
HFpEF: Heart Failure with a Preserved Ejection Fraction
HFrEF: Heart Failure with Reduced Ejection Fraction
RCT: Randomized Controlled Trial
